# ADDRESSING CAREGIVER TRANSPORTATION BARRIERS TO ENHANCE HEALTH CARE ACCESS FOR OLDER ADULTS

**DOI:** 10.1093/geroni/igae098.4109

**Published:** 2024-12-31

**Authors:** Renée St Louis, Nicole Zanier, Jennifer Zakrajsek, Colleen Peterson

**Affiliations:** University of Michigan Transportation Research Institute, Ann Arbor, Michigan, United States; University of Michigan Transportation Research Institute, Ann Arbor, Michigan, United States; University of Michigan Transportation Research Institute, Ann Arbor, Michigan, United States; University of Michigan Transportation Research Institute, Ann Arbor, Michigan, United States

## Abstract

Providing transportation assistance to an aging family member who no longer drives remains a pivotal caregiving task. Caregiver challenges related to coordinating and providing this transportation can have an impact on the frequency and level of health care an older adult can access. Applying the social ecological model, this qualitative study aimed to gain a contemporary, in-depth understanding of caregiver experiences facilitating health care access for those who no longer drive to inform health care solutions across various levels of the system. Transcribed audio recordings of four focus groups divided between urban and rural areas (N=21 individuals providing transportation assistance to aging adults) were iteratively reviewed to draw out themes to meet this aim. Participants described their health care transportation burden and barriers to health care access for their care recipients as well as their visions for more viable options and services. Participants expressed the need for streamlined care services including, for example, organization-based policy changes to improve safe transference from a personal or service vehicle and into the hospital/clinic. Perceived inadequacy of community transportation options led to participants filling the gaps by providing rides, asking friends for help, or posting on social networking sites to ensure health care access for their care recipients. On an interpersonal level, participants highlighted the benefits of intangible yet critical support, such as compassionate employees. Findings underscore multifaceted challenges caregivers face in providing transportation assistance, emphasizing the need for systemic improvements and policy changes to enhance health care access and support services.

